# Circadian Modulation of Behavioral Stress Responses in Zebrafish Is Age‐Dependent

**DOI:** 10.1002/jez.2905

**Published:** 2025-01-20

**Authors:** Santiago Pintos, Tyrone Lucon‐Xiccato, Luisa María Vera, Francisco Javier Sánchez‐Vázquez, Cristiano Bertolucci

**Affiliations:** ^1^ Department of Life Sciences and Biotechnology University of Ferrara Ferrara Emilia‐Romagna Italy; ^2^ Department of Physiology Faculty of Biology University of Murcia Murcia Region de Murcia Spain

**Keywords:** age‐related response, anxiety‐like behavior, circadian rhythm, fish welfare, stress

## Abstract

In the wild, stressors occur with varying likelihood throughout the day, leading animals to evolve plastic stress responses that exhibit circadian rhythmicity. In mammals, studies have revealed that the circadian plasticity of stress response may differ with age. However, such developmental effects have been largely overlooked in other vertebrate groups. In our research, we explored the presence of developmental variation in the daily pattern of behavioral stress response in a teleost fish model: the zebrafish (*Danio rerio*). We compared juvenile and adult individuals in two behavioral paradigms commonly used to analyze fish stress response, such as the open‐field test and the diving test. Our comparisons were conducted every 4 h during a 24‐h cycle to analyze daily variations. Significant daily rhythms were detected for almost all analyzed behaviors in both tests. In general, the analyses suggested a greater stress response in adults during the daytime and in juveniles during the night‐time, although not all indicators aligned in this direction. Moreover, we found average differences in zebrafish behavior, suggesting that juveniles were more sensitive to stress. Overall, these findings highlight the importance of considering developmental variation in the circadian pattern of stress response in non‐mammalian species like zebrafish.

## Summary

- Stress behavior is modulated by age and time of day in zebrafish
- Aligning husbandry practices with circadian rhythms and developmental stages could improve fish welfare

## 1 Introduction

Over the course of their lives, animals experience a wide variety of challenges that endanger their welfare and survival. Some of these stressors occur with different likelihood throughout the day (Favreau et al. 2009; Kolbe and Squires 2007; O'reilly, Armstrong, and Coleman 1986; Steiner, Leisch, and Hackländer 2014; Zielinski 1986), and consequently, animals have evolved behavioral responses that show a daily rhythm, allowing adaptive copying with the stressors (Bosiger and McCormick 2014; Fischer et al. 2019; Fraker 2008; Sönnichsen et al. 2013; Tolon et al. 2009; Voutilainen 2010; Watts et al. 2018). For instance, frog tadpoles repeatedly exposed to predation risk from salamanders in the evening displayed stronger antipredator responses in this phase of the day (Ferrari, Messier, and Chivers 2008). This behavioral adaptation is likely due to the daily variation in stress hormones commonly observed in several vertebrates (Allen‐Rowlands et al. 1980; Atkinson and Waddell 1997; Dunn, Scheving, and Millet 1972; Kramer and Sothern 2001; Lance and Lauren 1984).

Daily modulation of behavioral stress responses is also visible in standardized laboratory settings. For example, we recently reported behavioral differences in zebrafish, *Danio rerio*, exposed to the open‐field test at different times of the day (Pintos et al. 2023). The open‐field paradigm consists of exposing the subjects to a novel, empty environment, which is likely perceived as threatening and therefore, used to estimate stress responses (Cachat et al. 2010; Blaser, Chadwick, and McGinnis 2010; Stewart et al. 2012). Results of zebrafish studies indicated that behavioral stress responses were stronger during the dark phase compared to the light phase of the day (Manuel et al. 2014; Pintos et al. 2023).

Studies in several mammalian species have shown that the daily variations in stress responses may be different among individuals of different ages (Goncharova et al. 2008; Pardon et al. 2000; Van Cauter, Leproult, and Kupfer 1996). For example, young rats show greater and more prolonged physiological stress responses during the light phase compared to adults (Romeo et al. 2006; Romeo, Bellani, and McEwen 2005). The potential causes of such developmental effects are several. The circadian system might undergo developmental maturation and become more effective with increasing age (Allen and Kendall 1967). Alternatively, the challenges experienced by individuals of different ages may differ, leading to adaptive developmental differences in behavioral stress responses (Andersen 2003; Clutton‐Brock 1991; Romeo et al. 2016).

In non‐mammalian species, the interaction between age and circadian variation in behavioral stress responses has been little explored, although there is evidence suggesting this effect could occur in various taxa (*Gallus gallus*, Jiang, Fu, and Cheng 2023; *Drosophila melanogaster*, Kuintzle et al. 2017). In teleost fish, the impact of circadian time (Pintos et al. 2023; Thoré, Brendonck, and Pinceel 2021) and age (Kacprzak et al. 2017; Mariën et al. 2024; Vasconcelos et al. 2023) on behavioral stress responses has been investigated multiple times, as well as age‐related physiological stress responses (Auperin and Geslin 2008; Barcellos et al. 2012; Koakoski et al. 2012; Moreira et al. 2019). Nevertheless, to the best of our knowledge, age and circadian effects on behavior have not been investigated simultaneously.

In the present research, we aimed to investigate the interaction between age and circadian time on the behavioral stress response of zebrafish, a species that is often used as a model for translational neuroscience and behavioral research (Mrinalini et al. 2023). We focused on behavior as this ultimately determines how the individuals interact with the environment and cope with the stressors. Moreover, there is growing interest in behavioral stress indicators due to their applicability to practical conditions, such as the assessment of welfare in captive animals (Barreto et al. 2022; Martins et al. 2012). Adult and juvenile individuals were exposed to two well‐established behavioral paradigms for evaluating fish stress response, specifically the open‐field and diving tests (Blaser and Rosemberg 2012; Cachat et al. 2010; Godwin et al. 2012; Stewart et al. 2012). These tests were conducted at 4 h intervals throughout 24 h cycles with 12 h of light and 12 h of dark. We hypothesized that (1) the average stress response varies among age groups, (2) the stress response varies according to the time of day, and (3) the time‐of‐day impacts stress responses differently in the two age groups.

## 2 Materials and Methods

### 2.1 Experimental Subjects

Juvenile (1.5 months old; length: 0.98 ± 0.26 cm, *n* = 192) and adult (6–7 months old; length: 2.64 ± 0.27 cm, *n* = 192) wild‐type outbreed zebrafish were obtained from the fish facility of the University of Ferrara. For 1 month before the experiments, the fish were maintained separated by age in multiple 200‐L housing tanks (30–40 fish/tank; sex ratio 50:50) kept at constant temperature of 27 ± 1°C. This was done to ensure proper acclimation to the laboratory conditions and minimize confounds related to the transportation. All tanks were equipped with constant aeration and supplied with freshwater filtered by mechanical, chemical, and biological filters. The tanks were exposed to a 12:12 h light‐dark (LD) cycle with light on at 08:00 h (“zeitgeber” time 0; ZT0) and light off at 20:00 h (ZT12). Fish were fed twice daily with *Artemia salina* nauplii (at ZT1) and commercial fish pellets (Vipan Nature, Sera GmbH, Heinsberg, Germany) (at ZT8). The experimental subjects used in the experiments were randomly collected from the housing tanks to reduce as much as possible sampling biases (e.g., sex and size of the subjects). Behavioral experiments were conducted in a separate room from the housing tanks, where the fish were isolated from visual stimuli to reduce interference of external factors. Each fish was tested only once and was therefore naive. The videos from the adult fish tested in the open‐field test were re‐analyzed with a different time frame for another study (Pintos et al. 2023) due to ethical restrictions (i.e., to minimize the number of subjects involved in the research).

All experiments were conducted in accordance with the European Communities Council Directive of November 24, 1986 (86/609/EEC) and the law of the country in which they were performed (Italy, D.L. 4 Marzo 2014, n. 26). The Ethical Committee of University of Ferrara reviewed and approved all the experimental procedures (protocol n. CristianoBertolucci‐2019‐1 and LuconXiccato‐2022‐1).

### 2.2 Experiment 1—Open‐Field Test

One hundred ninety‐two subjects were tested overall in the open‐field test, 96 per each age group. Each subject was randomly assigned to one of the six testing time points (ZT2, 6, 10, 14, 18, 22). This resulted in six groups of 16 zebrafish tested every 4 h for each age group (*N* = 16 fish/ZT/age).

**FIGURE 1:**
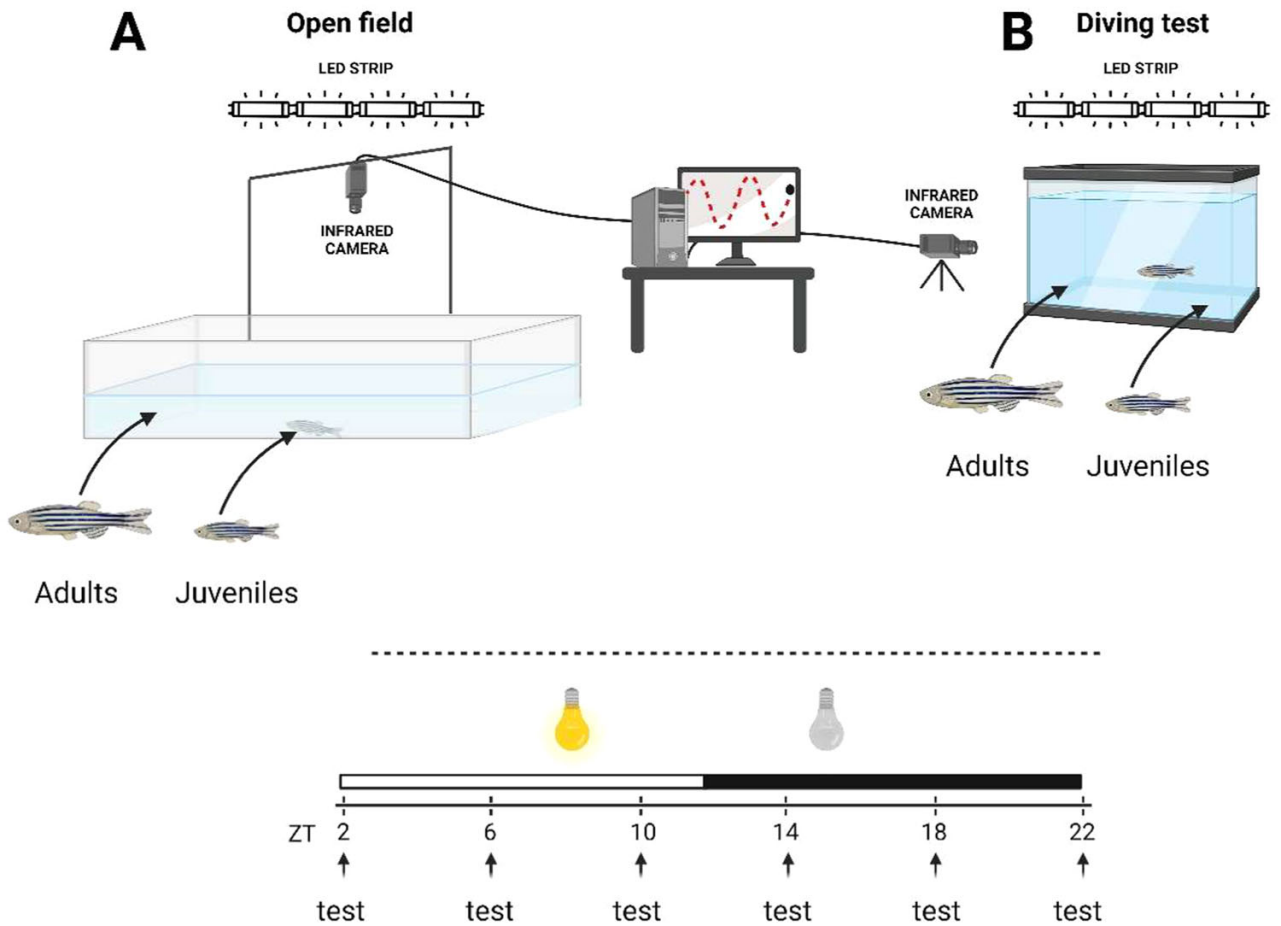
Diagram of the experimental design and testing apparatus. Juvenile and adult zebrafish were individually exposed to the open field test (A) and to the diving test (B) every 4‐h throughout a 24‐h. The experimental open field arena consisted of a white plastic rectangular tank of 40 × 40 × 8 cm for adults and 20 × 20 × 8 cm for juveniles. The diving test arena consisted of a glass rectangular tank of 20 × 6 × 20 cm for adults and 12 × 6 × 20 cm for juveniles. Each trial consisted of 6 min of novel environment exposure while different anxiety‐like behaviors were tracked by the Ethovision XT® software.

The open‐field testing was conducted in an empty, unfamiliar arena made of white plastic. The size of the arena was matched to the size of the two age groups. For the juveniles, 20 × 20 × 8 cm and filled with 2.5 L of water. For the adults, the arena was 40 × 40 × 8 cm and filled with 13 L of water. The water used in the test was dechlorinated tap water, changed between trials to avoid exposure to chemical cues of the previous subjects. The testing arenas were placed on a backlight table illuminated from below with infrared LEDs (λ > 980 nm; Noldus Information Technology, Wageningen, The Netherlands). The experimental room was kept in darkness during the experiments. However, a white LED strip (6500 K, Superlight Technology Co. Ltd., Shenzhen, China) illuminated the experimental arena from above during daytime (ZT0‐12). An infrared‐sensitive camera (Monochrome GigE camera, Basler, Germany; resolution: 1280 × 1024) was placed 1 m above the open field arena to record fish behavior (Figure 1A).

In each trial, one experimental subject was individually transported to the arena using an opaque jar, trying to minimize disturbance. Fish were then gently released in the center of the arena by inserting the jar in the water and slowly turning it. Then, the fish's behavior was recorded for 6 min while a computer running the EthoVision XT software (Noldus Information Technology, Wageningen, The Netherlands) live‐tracked it. A set of different behavioral indicators of stress were measured. The first was the time spent in the outer part of the arena (thigmotaxis) considering the center as a 20 × 20 cm sector for the adults and a 10 × 10 cm sector for the juveniles. This metric usually increases when zebrafish are stressed (Champagne et al. 2010). The second indicator was the time spent motionless (freezing) with a minimum speed threshold scaled according to fish body size (lower than 0.4 cm/s for the adults; and 0.15 cm/s for the juveniles; Pintos et al. 2023). Freezing is often linked with anxiety‐like states in zebrafish (Egan et al. 2009). The third indicator collected was the distance traveled (activity), usually considered a proxy for the arena exploration (Levin, Bencan, and Cerutti 2007). Last, angular velocity was recorded as an indicator of erratic movements, which generally represent evidence of stress when increased (Blaser, Chadwick, and McGinnis 2010).

### 2.3 Experiment 2—Diving Test

Ninety‐six naive zebrafish for each age group (i.e., juveniles and adults) were tested in the diving test. As described for experiment 1, these subjects were randomly assigned to one of the six testing times. This resulted in six groups of 16 fish tested every 4 h for each age group (*N* = 16 fish/ZT/age; N overall = 192 zebrafish).

The diving test arena consisted of a 2.4 L glass rectangular aquarium (20 × 6 × 20 cm) for adults and 1.4 L for juvenile fish (12 × 6 × 20 cm), filled with new dechlorinated tap water for each subject. The walls of the arena were opaque on three sides and transparent on the front side to allow recording of the swimming depth of the fish. The camera was located at 40 cm from the front of the experimental tank, while an infrared backlight panel was placed behind the arena (Figure 1B). A white LED strip (6500 K, Superlight Technology Co. Ltd., Shenzhen, China) illuminated the arena from above during the daytime (ZT0‐12). As described for experiment 1, fish were individually transported to the experimental room using an opaque jar and gently released in the center of the diving test arena. The tracking of the subjects was performed by the Ethovision XT software for 6 min after their release into the experimental arena. Three indicators collected in the previous test were recorded by the tracking software: freezing, activity and erratic movement. Moreover, the software recorded the time spent in the lower part of the arena (i.e., bottom dwelling). As the lower part of the arena, we considered a 20 × 10 cm sector for the adults and a 12 × 10 cm sector for the juveniles. The bottom dwelling latter parameter has been described as a robust stress indicator in zebrafish (Maximino et al. 2010; Tran and Gerlai 2016).

### 2.4 Statistical Analysis

Statistical analyses were conducted in R Statistical software version 4.0.1 (The R foundation for Statistical Computing Vienna Austria http://www.r-project.org). All descriptive statistics in the text represent mean ± standard error and the significance level of the tests was set at *p* = 0.05.

All the behavioral indicators measured were subjected to the Cosinor analysis to detect daily rhythms using the “Ritme” software (Prof. A. Díez‐Noguera, University of Barcelona, Spain). Cosinor analysis employs least‐squares regressions to model cosine curves, which are useful to describe circadian variations (Nelson et al. 1979). This analysis estimates a circadian rhythm through a zero‐amplitude test, in which *p* < 0.05 constitutes evidence for a statistically significant rhythm of the time interval under consideration (i.e., 24 h). Rhythm parameters such as mesor, acrophase and amplitude were calculated for each behavioral rhythm (Table 1). All acrophases were transformed from radians to time values (i.e., ZT). The statistical comparisons of acrophase and mesor were performed with the “*circa‐compare*” R package, which estimates differences in cosinor parameters by non‐linear regressions (Parsons et al. 2020).

**TABLE 1:** Cosinor parameters of behavioral indicators collected in the open field (OF) and diving tests (DT) obtained by Cosinor analysis.

Age group	Test	Behavior	Acrophase	*Significance*	Mesor	*Significance*
juvenile	OF	ACTIVITY	8.35 ± 2.41	*p* = 0.02	742.73 ± 60.19	*p* = 0.01
adult	OF	ACTIVITY	4.31 ± 2.49		1968.41 ± 134.19	
juvenile	OF	THIGMOTAXIS	10.85 ± 3.74	*p* < 0.01	86.45 ± 2.48	*p* < 0.01
adult	OF	THIGMOTAXIS	19.15 ± 4.63		90.21 ± 1.82	
juvenile	OF	FREEZING	21.40 ± 2.76	*p* < 0.01	7.67 ± 2.12	*p* < 0.01
adult	OF	FREEZING	6.37 ± 3.37		3.01 ± 1.70	
juvenile	OF	ERRATIC MOVEMENT	21.21 ± 3.16	*p* < 0.01	282.74 ± 17.91	*p* < 0.01
adult	OF	ERRATIC MOVEMENT	5.58 ± 3.60		78.84 ± 12.39	
juvenile	DT	ACTIVITY	6.43 ± 1.55	*—*	619.90 ± 41.09	*—*
adult	DT	ACTIVITY	—		—	
juvenile	DT	BOTTOM	3.17 ± 3.87	*p* = 0.05	50.00 ± 3.33	*p* < 0.01
adult	DT	BOTTOM	5.89 ± 1.30		56.51 ± 3.37	
juvenile	DT	FREEZING	20.00 ± 2.15	*p* < 0.01	2.78 ± 0.88	*p* < 0.01
adult	DT	FREEZING	3.49 ± 5.68		1.39 ± 0.42	
juvenile	DT	ERRATIC MOVEMENT	22.20 ± 2.76	*p* = 0.15	313.32 ± 37.01	*p* < 0.01
adult	DT	ERRATIC MOVEMENT	4.50 ± 2.67		128.56 ± 6.07	

*Note:* Data are presented as mean ± confidence interval (set at 95%) and acrophases in time values (ZT). P values are calculated by comparing Cosinor parameters using the *Circacompare* R function.

Additional one sample *t*‐tests were performed for the thigmotaxis and the time spent at the lower half of the arena to compare the observed values to chance level (i.e., 25% and 50%, correspondingly).

## 3 Results

### 3.1 Open‐Field Test

#### 3.1.1 Thigmotaxis

**FIGURE 2:**
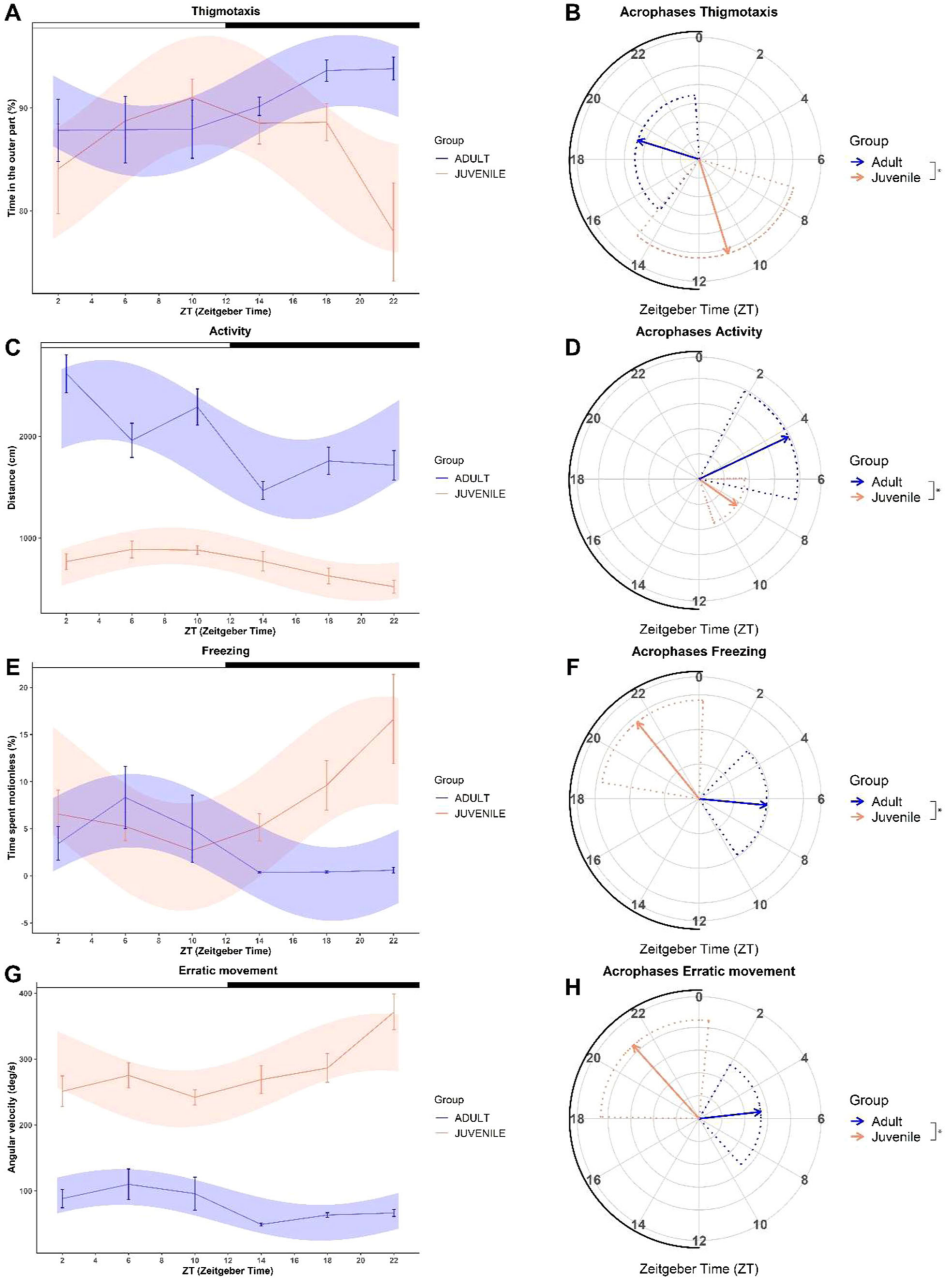
Results of the open‐field test. Observed values (linear plot: mean ± standard error) and values estimated from best cosinor fitting models (area plot) in adult (blue) and juvenile (orange) zebrafish for (A) thigmotaxis, (C) activity, (E) freezing and (G) erratic movement behaviors. White and black bars above each graph represent light and dark phases, respectively. Estimated acrophases for (B) thigmotaxis, (D) activity, (F) freezing and (H) erratic movement significant rhythms (Cosinor, *p* < 0.05) are represented in polarograms. Solid arrows indicate the mean acrophase and dotted lines indicate the confidence interval (set at 95%). The radial axis represents the time of the day (ZT) and the vector length represents the amplitude of the rhythm. The black line above radial axis represents the dark phase. Asterisks indicate statistical differences in the acrophase between age groups, estimated by non‐linear regression models (*Circacompare: p* < 0.05).

On average, juvenile zebrafish spent 86.47 ± 1.29% (mean ± standard error) of the testing time in the outer part of the open‐field arena, whereas adults did so for 90.21 ± 0.93% of the time. Both age groups spent significantly more time in the outer part of the arena than expected in the case of random movements (one sample *t‐*test, juveniles: *t*_94_ = 28.20, *p* < 0.01; adults: *t*_95_ = 42.84, *p* < 0.01). This implied the presence of the expected thigmotaxis response for both juvenile and adult zebrafish. The Cosinor analyses on thigmotaxis highlighted significant daily rhythms for both juvenile (Cosinor: *p* = 0.01) and adult zebrafish (Cosinor: *p* = 0.03; Figure 2A). Thigmotaxis acrophases significantly differed between age groups (*p*_*acrophase*_ < 0.01; Figure 2B), being located close to the end of the light phase for the juveniles (ZT = 10.85) and at the end of the dark phase for the adults (ZT = 19.15). The mesor was significantly different between age groups (*p*_*mesor*_ = 0.01), indicating that the adult zebrafish spent more time in the outer part of the arena compared to the juvenile.

#### 3.1.2 Activity

The average distance traveled by adults was 1968.41 ± 72.65 cm, whereas juvenile zebrafish traveled 744.28 ± 32.75 cm. Cosinor analysis showed significant daily rhythms of activity for both age groups (Cosinor: juveniles, *p* < 0.01; adults, *p* < 0.01; Figure 2C), evidencing significantly different acrophases throughout the light phase (juveniles: ZT = 8.35; adults: ZT = 4.31; *p*_*acrophase*_ = 0.02; Figure 2D). The mesor was significantly different between age groups (*p*_*mesor*_ < 0.01), indicating lower average activity values for juveniles.

#### 3.1.3 Freezing

Juvenile zebrafish exhibited freezing for an average of 7.63 ± 1.13% of the testing time. Adult zebrafish displayed freezing for 3.01 ± 0.89% of the testing time. The Cosinor analysis on freezing indicated significant daily rhythms for both juveniles (Cosinor: *p* < 0.01) and adults (Cosinor: *p* < 0.01; Figure 2E). The acrophases of these rhythms were located at the end of the dark phase for the juveniles (ZT = 21.40) and in the middle of the light phase for the adults (ZT = 6.37), with a significant difference between age groups (*p*_*acrophase*_ < 0.01; Figure 2E). The mesor was also significantly different between age groups (*p*_*mesor*_ < 0.01), indicating greater average freezing values for juveniles.

#### 3.1.4 Erratic Movement

On average, the angular velocity of adult zebrafish was 78.84 ± 6.48 deg/s and that of juvenile zebrafish was 282.44 ± 9.46 deg/s. Daily rhythms were detected for the erratic movements of both juveniles (Cosinor, *p* < 0.01) and adults (Cosinor, *p* = 0.01; Figure 2G), with a significant difference in the acrophase between age groups (juveniles: ZT = 21.21; adults: ZT = 5.58; *p*_*acrophase*_ = 0.02; Figure 2H). The mesor was significantly different between age groups (*p*_*mesor*_ < 0.01), indicating greater average erratic movement values for juveniles.

### 3.2 Diving Test

#### 3.2.1 Bottom Dwelling

**FIGURE 3:**
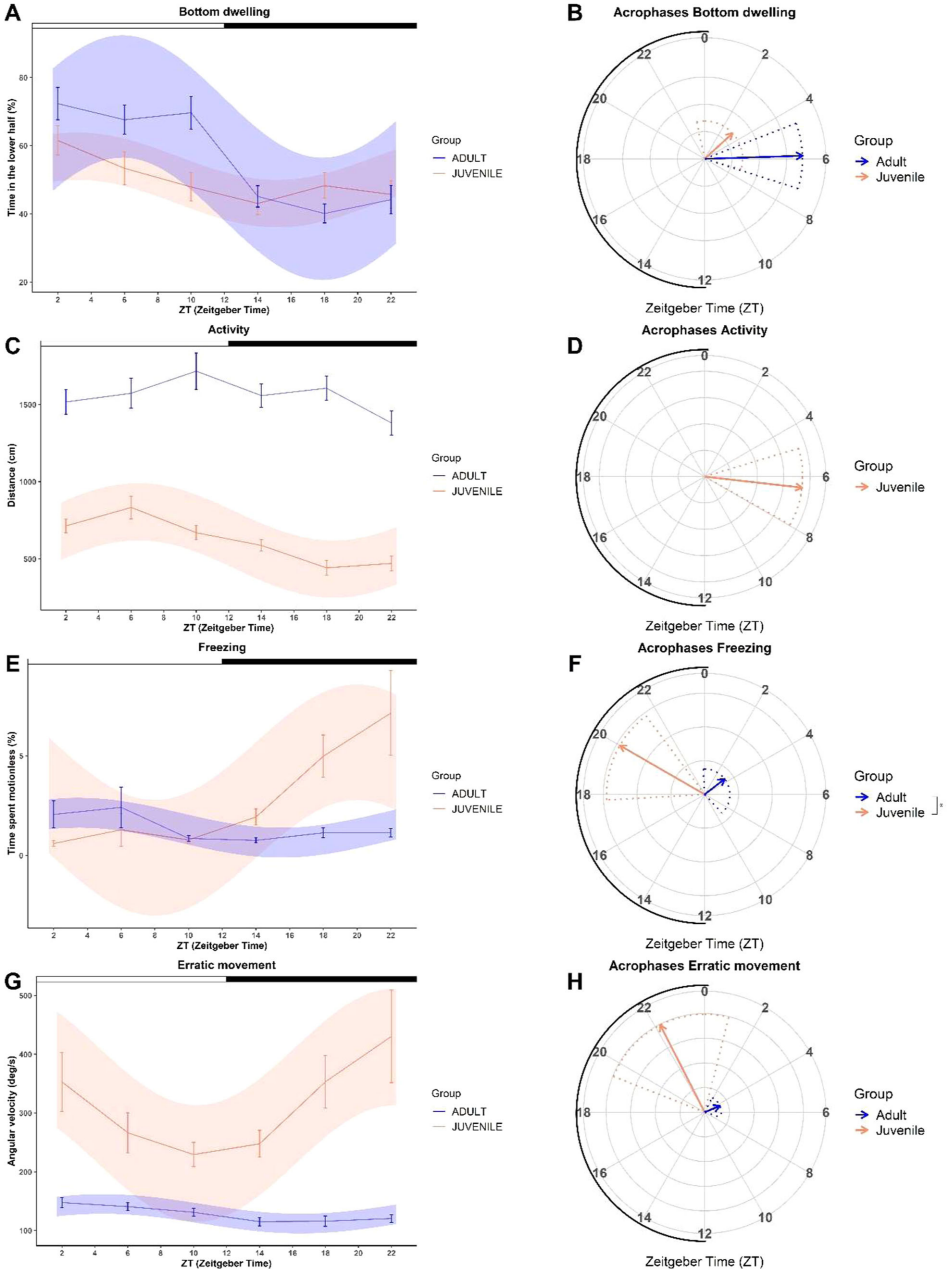
Results of the diving test. Observed values (linear plot: mean ± standard error) and values estimated from best cosinor fitting models (area plot) in adult (blue) and juvenile (orange) zebrafish for (A) bottom dwelling, (C) activity, (E) freezing and (G) erratic movement behaviors. White and black bars above each graph represent light and dark phases, respectively. Estimated acrophases for (B) bottom dwelling, (D) activity, (F) freezing and (H) erratic movement significant rhythms (Cosinor, *p* < 0.05) are represented in polarograms. Solid arrows indicate the mean acrophase and dotted lines indicate the confidence interval set at 95%. The radial axis represents the time of the day (ZT) and the vector length represents the amplitude of the rhythm. The black line above radial axis represents the dark phase. Asterisks indicate statistical differences in the acrophase between age groups, estimated by non‐linear regression models (*Circacompare*: *p* < 0.05).

Juvenile zebrafish spent an average of 50.00 ± 1.73% of testing time in the lower sector, a response that did not differ from that expected by chance for random movements (one sample *t‐*test: *t*_*95*_ < 0.01, *p* = 0.99). Conversely, adults spent an average of 56.51 ± 2.12% of the testing time in the lower sector, denoting higher values than those expected by chance (*t*_*95*_ = 3.06, *p* < 0.01). Daily rhythms were found for both juvenile (Cosinor, *p* = 0.01) and adult zebrafish (Cosinor, *p* < 0.01; Figure 3A). The acrophases of the two age groups were located in the light phase (juveniles: ZT = 3.16; adults: ZT = 5.88) and were not significantly different (*p*_*acrophase*_ = 0.05; Figure 3B). The mesor was significantly different between age groups (*p*_*mesor*_ < 0.01), indicating more time spent in the lower half of the arena for adults.

#### 3.2.2 Activity

The activity observed was on average 619.89 ± 24.51 cm in juveniles and 1558.43 ± 36.90 cm in adults. The Cosinor analysis found significant daily rhythms in activity for juveniles (Cosinor, *p* < 0.01), but not for adults (Cosinor, *p* = 0.08; Figure 3C). Juveniles' acrophase was located close to the middle of the light phase, at ZT = 6.43. Due to the absence of significant rhythmicity in the adult activity, it was not possible to compare the parameters of acrophase and mesor between age groups (Figure 3D).

#### 3.2.3 Freezing

Juveniles and adults displayed freezing for 2.78 ± 0.48% and 1.39 ± 0.21% time, respectively. The Cosinor analysis revealed significant daily rhythms in freezing behavior for both age groups (juveniles: Cosinor, *p* < 0.01; adults: Cosinor, *p* = 0.04; Figure 3E). The acrophase of the juveniles was located in the dark phase (ZT = 20.00) and that of adult fish was located in the light phase (ZT = 3.48), with a significant difference between the two age groups (*p*_*acrophase*_ < 0.01; Figure 3F). The mesor was also significantly different between age groups (*p*_*mesor*_ < 0.01), indicating higher freezing in juveniles.

#### 3.2.4 Erratic Movement

Juvenile zebrafish displayed an average angular velocity of 313.32 ± 19.79, while adult of 128.56 ± 3.25 deg/s. The Cosinor analysis revealed daily rhythms in erratic movements for both age groups (Cosinor, *p* < 0.01; Figure 3G). Although acrophases were located at different phases of the cycle in adults (ZT = 4.50) and juveniles (ZT = 22.20), significant differences in this parameter were not detected between age groups (*p*_*acrophase*_ = 0.15; Figure 3H). The mesor was significantly different between age groups (*p*_*mesor*_ < 0.01; Figure 3H), indicating greater erratic movement in the juveniles.

## 4 Discussion

This study examined the stress response of juvenile and adult zebrafish through open‐field and diving tests, assessing behavioral indicators of stress across the 24 h cycle. Our analyses showed that both age groups exhibited consistent daily variations in almost all stress‐related behaviors. Notably, the impact of time of day differed significantly between juveniles and adults.

In both assays, the cosinor analysis generally detected daily rhythms in behavior for juvenile and adult zebrafish (i.e., in 15 out of the 16 variables considered). In fish, a large body of literature has similarly reported daily rhythms in a wide range of behaviors such as feeding (Di Rosa et al. 2024; López‐Olmeda and Sánchez‐Vázquez 2010; Montoya et al. 2010; Vera et al. 2013), locomotion (Fortes‐Silva et al. 2010; del Pozo, Sánchez‐Férez, and Sánchez‐Vázquez 2011), and reproductive activities (Oliveira and Sánchez‐Vázquez 2010; Weber and Spieler 1987). Despite abundant physiological evidence of daily variations in fish stress responses (Cowan, Azpeleta, and López‐Olmeda 2017; López‐Olmeda et al. 2013), few studies have reported circadian effects on stress‐related behaviors (Pintos et al. 2023; Thoré, Brendonck, and Pinceel 2021). All behaviors analyzed in this study have been previously reported as stress indicators in zebrafish (Blaser, Chadwick, and McGinnis 2010; Champagne et al. 2010; Godwin et al. 2012; Maximino et al. 2010), and some of them have often been shown to covary with physiological stress markers (Archard et al. 2012; Egan et al. 2009; Lara and Vasconcelos 2021). Therefore, our findings contribute to this body of knowledge, suggesting that stress behavior in zebrafish is governed by the circadian clock.

In all the indicators collected in the open‐field test and in one indicator of the diving test, significant age‐related differences in the acrophase were observed. This supports our prediction that age influences the circadian modulation of behavioral responses to stress in fish. Given that similar age‐related differences in circadian modulation have been observed in various other vertebrate species (Goncharova et al. 2008; Romeo et al. 2006; Van Cauter, Leproult, and Kupfer 1996), it is plausible that this phenomenon is common among animals, perhaps due to shared physiological mechanisms. However, understanding the underlying causes of this phenomenon requires further research. Indeed, they could involve both adaptive developmental changes or processes of maturation of the circadian clock system (Dekens and Whitmore 2008; Vatine et al. 2011). It is worth noting that age differences in the daily pattern were more evident in the indicators collected from the open‐field test. The literature suggests that certain behavioral tests in zebrafish are more suitable than others for investigating specific effects (Blaser, Chadwick, and McGinnis 2010; Colchen et al. 2017; Dereje et al. 2012). This is possibly due to the characteristics of each test, such as the size and shape of the experimental arena, and even the lighting conditions, which have been shown to significantly influence behavior assessment in fish (de Abreu et al. 2020; Hodorovich et al. 2024; Lovin et al. 2023). Based on our study, we recommend using the open‐field test for examining behavioral stress responses due to its higher sensitivity in detecting age‐related circadian variations, while the diving test might have limitations for this purpose.

Interestingly, we did not find a complete agreement in the direction of age modulation of daily stress across the different indicators. For freezing and erratic swimming in the open‐field test and for freezing in the diving test, analyses indicated acrophases in the light phase for adults and during the dark phase for juveniles. The same pattern, though not significant, was found in the erratic movement indicator collected in the diving test, possibly due to the aforementioned limitations of the diving test for these applications. Additionally, activity in the open‐field test followed this pattern: while both age groups had acrophases during the light phase, juveniles' acrophase occurred significantly later. The aforementioned four indicators suggest greater stress responses during the light phase for adults and during the dark phase for juveniles. However, one indicator seems to deviate from this pattern: thigmotaxis in the open‐field test was greater during the dark phase for adults, as previously observed in Pintos et al. (2023), and instead greater at the end of the light phase in juveniles. This suggests greater stress occurring during the night in adults and during the day in juveniles. The reason for this age‐related discrepancy is unclear at this stage of research. While thigmotaxis is quite used in adult zebrafish and other teleost species (Godwin et al. 2012; Pintos et al. 2024; Watanabe et al. 2021), two studies found that immature zebrafish displayed thigmotaxis when exposed to a sudden distressing stimulus during the test (Schnörr et al. 2012a; Schnörr et al. 2012b). Since we did not use this type of stimulation, our test might not have been sufficient to trigger the typical thigmotaxis response in juveniles. Moreover, consistent with the general difficulties in comparing this indicator between zebrafish of different ages, another study found that individual differences in thigmotaxis are not consistent across life stages in this species (Alfonso et al. 2020). This suggests that thigmotaxis might be a less reliable indicator of stress across different developmental stages in zebrafish, highlighting the need for further research to understand its variability and underlying mechanisms (e.g., stress hormones).

Overall, although we acknowledge the need for confirmation due to the results of thigmotaxis, most of the evidence suggests that adult fish might exhibit greater stress responses during the day, while juvenile fish might do so during the night or at least towards the end of the day. If confirmed, this finding could significantly impact fish welfare practices. For instance, it would inform more appropriate timing for stressful manipulations based on the age of the fish, enhancing welfare protocols by aligning with their circadian stress responses. Moreover, our research paves the way for investigations into the potential ecological causes of the observed age differences. For instance, it might be possible that juvenile zebrafish experience greater predation risk during the night compared to the day (Bosiger and McCormick 2014). Understanding these ecological factors could provide deeper insights into the adaptive significance of the circadian modulation of stress responses in zebrafish and other species.

A secondary finding of our work is the presence of average differences in stress responses across age groups. As evidenced by the mesor comparisons, juveniles displayed stronger responses compared to adults in five out of six significant tests. The one exception was the diving test measure of time spent at the bottom, where we found the opposite pattern. However, an earlier study suggests that this discrepancy was due to a change in swimming depth preference across ages in zebrafish rather than a real change in stress (Péan et al. 2013). Despite the fact that this behavior is generally considered a robust indicator of stress in the diving test in a wide range of teleost (Cachat et al. 2010; Pintos et al. 2021; Tran and Gerlai 2016; Zhang et al. 2023), our results may be biased by age‐related depth preferences. Therefore, it seems likely that juveniles suffer stronger stress compared to adults, a finding that has been reported in earlier studies as well (Mariën et al. 2024; Henríquez Martínez et al. 2022; Polverino et al. 2016).

In conclusion, this research sheds light on how stress behavior is modulated by age and time of day in a model teleost species. Our findings provide insights for better assessing fish stress responses to improve fish welfare. Given the growing trend and widespread application of behavioral indicators to non‐invasively assess fish welfare (Barreto et al. 2022; Cavallino, Rincón, and Scaia 2023), it becomes crucial to control these factors (age and time of day) to mitigate data biases and ensure the reliability of behavioral outcomes. Moreover, proper consideration of these factors is required when manipulating fish during maintenance procedures, such as in aquaculture, to ultimately safeguard fish welfare. Further studies should focus on correlating behavioral indicators of stress with physiological (i.e., cortisol) and molecular (i.e., hypothalamic‐pituitary‐interrenal axis genes) markers to provide a more comprehensive understanding of the underlying mechanisms and to further enhance the accuracy and reliability of fish welfare assessments.

## Author Contributions

**Santiago Pintos:** conceptualization, methodology, formal analysis, investigation, data curation, writing–original draft. **Tyrone Lucon‐Xiccato:** formal analysis, data Curation, writing–review and editing. **Luisa María Vera:** conceptualization, writing–review and editing, supervision. **Javier Sánchez‐Vázquez:** writing–review and editing, supervision, project administration, funding acquisition. **Cristiano Bertolucci:** conceptualization, resources, writing–review and editing, supervision, project administration, funding acquisition.

## Data Availability

The data that support the findings of this study are available from the corresponding author upon reasonable request.
